# Fibrinogen as a Prognostic Predictor in Pediatric Patients with Sepsis: A Database Study

**DOI:** 10.1155/2020/9153620

**Published:** 2020-04-28

**Authors:** Xiaomeng Tang, Lujing Shao, Jiaying Dou, Yiping Zhou, Min Chen, Yun Cui, Yucai Zhang, Chunxia Wang

**Affiliations:** ^1^Department of Critical Care Medicine, Shanghai Children's Hospital, Shanghai Jiao Tong University, Shanghai 200062, China; ^2^Institute of Pediatric Critical Care, Shanghai Jiao Tong University, Shanghai 200062, China; ^3^Department of Information Technology, Shanghai Children's Hospital, Shanghai Jiao Tong University, Shanghai 200062, China

## Abstract

**Background:**

Systemic inflammatory response and vascular endothelial cell injury during sepsis lead to coagulopathy. Fibrinogen has been reported as a biomarker of coagulopathy; however, the prognostic value of fibrinogen remains undefined in pediatric patients with sepsis. The aim of this study was to assess fibrinogen level on pediatric intensive care unit (PICU) admission and to elucidate the relationship between fibrinogen levels and in-hospital mortality in children with sepsis.

**Methods:**

We conducted a database study. The sepsis database was divided into a training set (between July 2014 and June 2018) and a validation set (from July 2018 to June 2019). The clinical and laboratory parameters on PICU admission and in-hospital mortality in sepsis database were collected and analyzed.

**Results:**

A total of 819 pediatric patients were included from database as a training set. The overall hospital mortality was 12.1% (99/819). The fibrinogen levels were significantly lower in nonsurvivors than survivors. Multivariate *logistic* regression analysis showed significant associations between fibrinogen, lactate level, and hospital mortality (fibrinogen: *odds ratio* (OR), 0.767 (95% CI: 0.628-0.937), *P* = 0.009; lactate: OR, 1.346 (95% CI: 1.217-1.489), *P* < 0.001, respectively), which was confirmed in a validation set (0.616 [95% CI: 0.457-0.829], *P* = 0.001; 1.397 [95% CI: 1.245-1.569], *P* < 0.001, respectively). The hospital mortality of patients with fibrinogen < 1 g/L, 1-2 g/L, 2-3 g/L, or over 3 g/L displayed an obvious difference (62.5% *vs.* 27.66% *vs.* 18.1% *vs.* 4.2%, respectively). Furthermore, the area under the receiver operating characteristic curve (ROC) for fibrinogen in predicting hospital mortality was 0.780 (95% CI: 0.711-0.850) in pediatric patients with sepsis.

**Conclusions:**

Fibrinogen is a valuable prognostic biomarker for pediatric sepsis. The level of fibrinogen lower than 2 g/L on PICU admission is closely related to the greater risk of hospital death in pediatric sepsis.

## 1. Introduction

Sepsis is the main cause for nonselective admission to the pediatric intensive care unit (PICU). The incidence of sepsis was 3.0 million cases in neonates and 1.2 million cases in children annually, and overall mortality rate was ranged from 9 to 20% for severe sepsis worldwide [1]. Routine clinical and laboratory detection would be done for each patient on PICU admission. However, it is still challenging for clinicians how to assess the prognosis of patients with sepsis based on these available data. Until now, there is lack of specific or sensitive index to predict the outcome of sepsis, particularly in the early period of sepsis.

Sepsis-induced coagulopathy is a hallmark of sepsis, which leads to widespread thrombosis in the microvasculature. Accumulated studies indicated that coagulopathy is associated with sepsis-associated organ failure and mortality [2–4]. Fibrinogen is a biomarker for diagnosis of coagulopathy in critically ill patients [5]. Recent report demonstrated that a fibrinogen level of approximately <200 mg/dL is associated with sharply increased mortality in adult patients with severe sepsis [6]. Moreover, neonates with sepsis who died displayed a lower level of plasma fibrinogen than survivors [7]. Hypofibrinogenemia is distinctive for a high mortality rate in septic children [8]. Though hypofibrinogenemia is clearly correlated with mortality in adults or neonates with sepsis, the prognostic value of hypofibrinogenemia in pediatric patients with sepsis in a large population is currently unavailable.

In this study, we analyzed the database based on 5-year medical records of patients with sepsis in a tertiary pediatric hospital. The aim of the study was to assess the value of the first detection of fibrinogen level on PICU admission and to clarify the association between fibrinogen level and hospital mortality in pediatric patients with sepsis.

## 2. Materials and Methods

### 2.1. Patients

We analyzed the sepsis database from the PICU at Shanghai Children's Hospital in a 5-year period. Patients with sepsis were diagnosed within 24 h after PICU admission based on the International Pediatric Sepsis Consensus Conference in 2005 [9]. We divided the database into a training set (between July 2014 and June 2018) and a validation set (from July 2018 to June 2019). In the training set, the inclusion criteria included (1) patients admitted to PICU with sepsis and (2) aged with over 28 days to 18 years old. The exclusion criteria included (1) without data about coagulation markers and (2) patients with primary liver disease, tumor including solid tumor and hematological malignancy, or heredity metabolic disease. In the validation set, we enrolled all patients with sepsis with the value of fibrinogen. Complete items of sepsis database include demographic, clinical, and laboratory parameters, as well as main treatment and outcome. The study was approved by the ethics committee of Children's Hospital affiliated to Shanghai Jiao Tong University (Approval number 2018R039-F01). Requirement for consent was waived as the data were recorded anonymously in the database.

### 2.2. Observational Variables

Data on all patients were obtained through a computerized database of all in-patient records of patients diagnosed with sepsis. We collected clinical parameters including age, gender, PRISM III score, and complications including respiratory failure, shock, gastrointestinal disorder, liver injury, acute kidney injury, heart failure, immune disorder, and brain injury. The laboratory parameters were determined in a clinical lab at Shanghai Children's Hospital, which included biochemical parameters of organ functions (alanine aminotransferase (ALT), direct bilirubin (DBIL), total bilirubin (TBIL), *γ*-glutamyl transpeptidase (*γ*-GT), lactate dehydrogenase (LDH), blood urea nitrogen (BUN), creatinine (Cr), lactate (Lac), and creatine kinase isoenzyme MB (CK-MB)), coagulation function (prothrombin time (PT), international normalized ratio (INR), fibrinogen (Fib), and activated partial thromboplastin time (APTT)), infectious parameters (C-reactive protein (CRP), white blood cell (WBC)), and platelet (PLT) count. The level of fibrinogen was determined by the clot-weight method. The outcome variables included the length of hospital stay and discharged survival status. The laboratory parameters were collected based on the first batch of data after PICU admission.

### 2.3. Statistical Analyses

Data analyses were performed using STATA 15.0MP (College Station, Texas, USA). Continuous variables were summarized as median (interquartile range (IQR)) of the abnormal distribution data. The Mann-Whitney *U* test was used to compare the medians of continuous variables with abnormal distribution data. The *chi*-squared test was used to compare the categorical data. Subgroup analysis was performed according to age or fibrinogen levels. Unadjusted associations between covariates and hospital mortality were estimated by bivariate *logistic* regression models. Adjusted *odds ratios* (ORs) and 95% *confidence intervals* (CI) were calculated using multivariate logistic regression analyses. Receiver operating characteristic curve (ROC) analysis was used to assess the efficiency of fibrinogen as a prognostic predictor in pediatric patients with sepsis. A value of *P* < 0.05 was considered statistically significant. All *P* values presented are two-tailed.

## 3. Results

### 3.1. Baseline Characteristics of Patients in the Training Set

**Figure 1:**
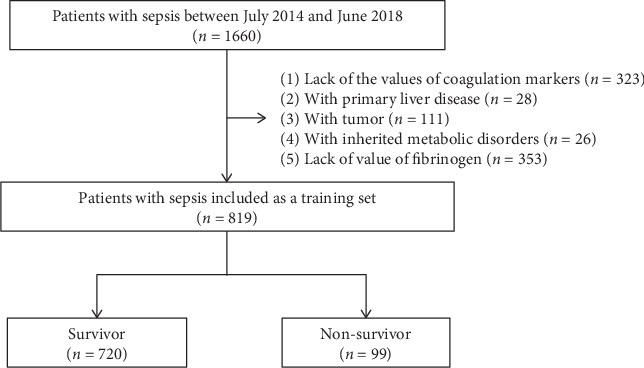
Flowchart of patients' selection in the training set.

In the training set, among the 1660 eligible patients, 323 cases were excluded due to lack of the values of coagulation markers, and patients with primary liver disease (28 cases), tumor (111 cases), or heredity metabolic disease (26 cases) were excluded. Lack of the value of fibrinogen (353 cases) was also excluded. Finally, 819 visits left were available for analyses in the training set (Figure 1). Patient characteristics of the training set were stratified according to survival status on hospital discharge (Table 1).

In this cohort, the hospital mortality rate was 12.1% (99/819). The median (IQR) age of pediatric patients on PICU admission was 26.2 (8.9, 64) months. There was no significant difference between survivor and nonsurvivor (*P* = 0.479). Most patients were male (61.7%). The median (IQR) length of hospital stay was 13 (9, 21) days in the survivor group and 8 (4, 21) days in the nonsurvivor group (*P* < 0.001). In the training set, the incidence rate of respiratory failure was 34.4% (248/720) in the survivor group, which was significantly lower than that in nonsurvivor group (66.7%, 60/99). There were 104 cases with shock (14.4%) in survivors, which was lower than in nonsurvivors (34.3%, 34/99) (*P* < 0.001). The incidence rates of gastrointestinal dysfunction, liver injury, acute kidney injury, heart failure, and brain injury were significantly different between survivors and nonsurvivors (all *P* < 0.05) (Table 1). In addition, the rates of extracorporeal life support including artificial respiration (*P* < 0.001), continuous renal replacement therapy (CRRT) (*P* < 0.001), or extracorporeal membrane oxygenation (ECMO) support (*P* = 0.012) were significantly higher in nonsurvivors than in survivors.

### 3.2. Fibrinogen Is Correlated with Lactate Levels

**Figure 2:**
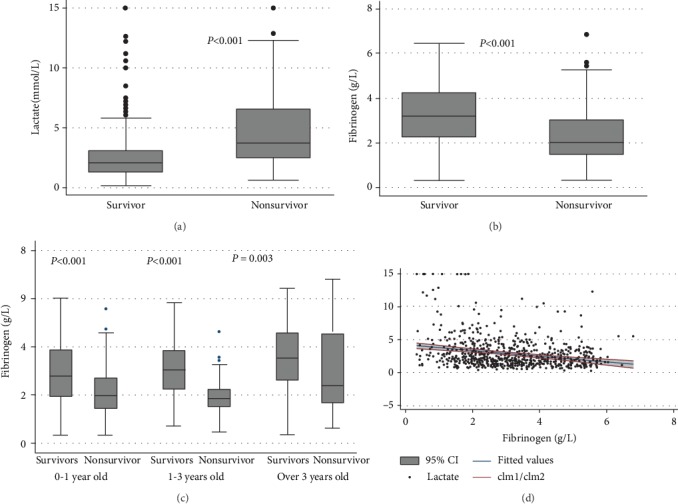
Levels of lactate and fibrinogen levels on PICU admission in patients with sepsis. (a) Lactate levels in survivors and nonsurvivors; (b) fibrinogen levels in survivors and nonsurvivors; (c) fibrinogen levels in children 0-1 year old, 1-3 years old, or over 3 years old; (d) correlation of fibrinogen with lactate levels.

Laboratory parameters of patients with sepsis were collected based on the first data on PICU admission and stratified according to survival status on hospital discharge (Table 2). Compared with survivors, nonsurvivors had significantly higher lactate levels (3.7 [2.5, 6.6] mmol/L *vs.* 2.05 [1.3, 3.1] mmol/L, *P* < 0.001; Figure 2(a)), and the fibrinogen levels were significantly lower in nonsurvivors than in survivors (2.02 [1.48, 3.06] g/L *vs.* 3.2 [2.27, 4.25] g/L, *P* < 0.001; Figure 2(b)). Subgroup analysis according to age indicated nonsurvivors displayed low fibrinogen level on PICU admission compared with survivors in children 0-1 year old, 1-3 years old, or over 3 years old (*P* < 0.001, *P* < 0.001, and *P* = 0.003, respectively; Figure 2(c)). Furthermore, the levels of fibrinogen were correlated with lactate levels (Figure 2(d)).

### 3.3. Multivariate Logistic Regression Analysis of Risk Factors for Hospital Mortality in Patients with Sepsis

The levels of Lac, PT, Fibrinogen, BUN, and PLT were significantly different between nonsurvivors and survivors on PICU admission (all *P* < 0.001) (Table 3). In the training set, univariate *logistic* regression analysis showed that lactate, PT, APTT, fibrinogen, DBIL, *γ*-GT, BUN, and PLT were significantly associated with hospital mortality (all *P* < 0.05), and multivariate *logistic* regression analysis indicated that lactate and fibrinogen were independent factors related to the mortality of pediatric sepsis (1.346 [1.217, 1.489], *P* < 0.001; 0.767 [0.628, 0.937], *P* = 0.009, respectively) (Table 3). Furthermore, the significance of both lactate and fibrinogen was confirmed in a validation set (1.274 [1.126, 1.442], *P* < 0.001; 0.619 [0.435, 0.88], *P* = 0.008, respectively) (Table 3).

### 3.4. Confirmation of the Value of Fibrinogen as Prognostic Predictor in Pediatric Sepsis in a Validation Set

**Figure 3:**
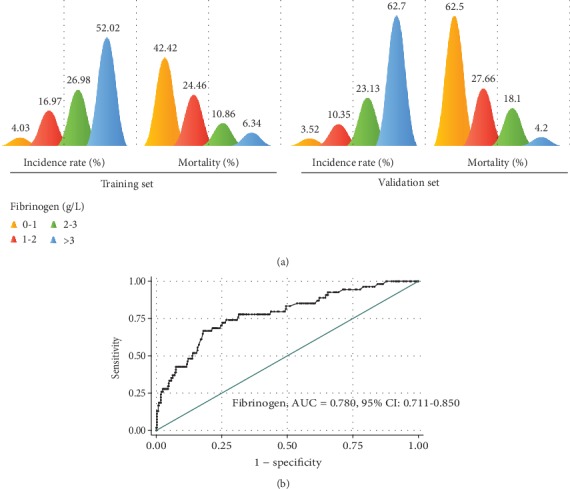
Fibrinogen as a prognostic predictor for hospital mortality. (a) The incidence and mortality rate in pediatric patients with different levels of fibrinogen (fibrinogen level: yellow: 0-1 g/L; red: 1-2 g/L; green: 2-3 g/L; and blue: >3 g/L) in the training set (left) and the validation set (right). (b) ROC analysis of fibrinogen as a prognostic predictor for hospital mortality in the validation set.

To confirm the relationship between fibrinogen levels on PICU admission and hospital mortality of sepsis, pediatric patients with sepsis admitted to PICU in another 1-year period were included as a validation set. All these patients were transferred from surgical department (5.1%, 23/454), emergency (76%, 345/454), and others (21.4%, 97/454). The main complications in these patients during PICU hospitalization included respiratory failure (48.2%, 219/454), shock (27.3%, 124/454), immune disorder (20.7%, 94/454), liver injury (20.7%, 94/454), and capillary leak syndrome (20.3%, 92/454) (Table 4). The ratio of artificial respiration (*P* < 0.001), CRRT (*P* < 0.001), or extracorporeal membrane oxygenation (ECMO) support (*P* = 0.033) was higher in nonsurvivor compared with survivors (Table 4). According to the levels of fibrinogen, the patients were divided into four subgroups. The ratios of patients with fibrinogen of below 1 g/L, 1-2 g/L, 2-3 g/L, or over 3 g/L on PICU admission were 4.03%, 16.97%, 26.98%, and 52.02% in the training set and 3.52%, 10.35%, 23.13%, and 62.7% in the validation set, respectively (Figure 3(a)). In the subgroup of patients with fibrinogen below 1 g/L on PICU admission, the mortality rate was 62.5% (10/16), 27.7% in the subgroup of patients with fibrinogen between 1 g/L and 2 g/L, 18.1% in the subgroup of patients with fibrinogen between 2 g/L and 3 g/L, and 4.2% in the subgroup of patients with fibrinogen over 3 g/L (Figure 3(a)). Furthermore, the area under the ROC curve (AUC) of fibrinogen for hospital mortality was 0.780 (95% CI: 0.711-0.850). The cutoff level of fibrinogen for hospital mortality is 2.46 g/L on PICU admission with sensitivity of 66.67% and specificity of 82.00% (Figure 3(b)).

**Figure 4:**
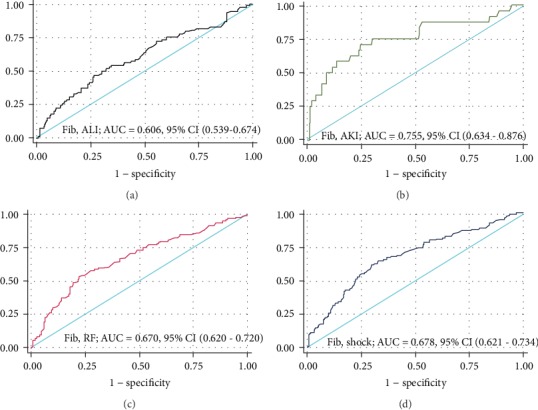
ROC analysis of fibrinogen as a predictor for sepsis-associated organ dysfunction in the validation set: (a) sepsis-associated acute liver injury (ALI); (b) sepsis-associated acute kidney injury (AKI); (c) sepsis-associated respiratory failure (RF); (d) septic shock (shock).

Furthermore, fibrinogen is a predictor for sepsis-associated AKI (AUC: 0.755 (0.634-0.876)), sepsis-associated respiratory failure (AUC: 0.670 (0.620-0.720)), septic shock (AUC: 0.678 (0.621-0.734)), or sepsis-associated liver injury (AUC: 0.606 (0.539-0.674)) (Figure 4 and Table 5).

## 4. Discussion

It is still a challenge for clinicians to assess the outcome of sepsis at the moment of PICU admission. To our knowledge, it is the first report based on database study to analyze the association between fibrinogen level and hospital mortality in pediatric patients with sepsis. Our results indicated that fibrinogen is a valuable prognostic biomarker for pediatric sepsis.

Previous study reported that lower level of plasma fibrinogen was detected in neonates who died, and plasma fibrinogen was proved to be an effective tool in assessing development of outcome in neonates [7]. Moreover, a dramatic increase in mortality was reported in patients with fibrinogen below 200 mg/dL in adult patients with severe sepsis [6]. Matsubara et al. found that the decrease of fibrinogen was independently associated with the increased risk of in-hospital mortality [6]. Consistently, the fibrinogen level on PICU admission was significantly lower in nonsurvivors than in survivors in our study. In our study, the mortality rate was gradually increased in response to decreased levels of fibrinogen. It is well known that increased coagulant activity and decreased fibrinolysis induced by inflammation lead to fibrin deposition in the microcirculation which contributes to organ dysfunction [10, 11]. Moreover, severe coagulopathy and/or DIC induce increased mortality in septic patients [10]. The DIC score at admission, but not prothrombin time (PT) and D-dimer, was proved to be associated with prognosis of sepsis in adult patients [12]. In our study, PT, APTT, and INR displayed significant differences between survivors and nonsurvivors, but none of these parameters were independent risk factors for hospital mortality in pediatric patients with sepsis. So, we suspected that the value of fibrinogen in prognostic prediction is partially related to the role of fibrinogen in coagulopathy. Generally, fibrinogen is a marker indicating the depletion of hemostatic factors and reflecting the excessive hypercoagulation and hyperfibrinolysis state in sepsis-induced coagulopathy, which is considered to be an acute-phase reactant which is typically increased in patients with infection and/or inflammation [13]. Fibrinogen remains in higher levels until the late stage of disease progression [4]. So, hyperfibrinogen at early phase of sepsis could reflect the adaptation of infection, which might contribute to early recovery from sepsis. Conversely, hypofibrinogen reflects the combination of consumption via microthrombosis and synthesis impairment in the liver, which implies the worse outcome of sepsis.

Serum lactate is widely used to assess the outcome of sepsis, and hyperlactatemia is a stronger predictor of mortality, and this risk rises exponentially with increase in lactate levels [14–16]. It was reported that lactate within 1 h of ICU admission was closely related to the risk of sepsis mortality in children [17]. In the present study, serum lactate was associated with the hospital mortality in pediatric patients with sepsis. Otherwise, CRP is a well-known parameter for diagnosis of sepsis or assessment of predictor of successful antibiotic therapy [18, 19]. Recent study indicated that on the 2^nd^, 3^rd^, and 5^th^ days, serum CRP level was higher in the nonsurvivor group than in the survivor group, and serum CRP has good clinical prognostic value for patients with sepsis and septic shock [20]. Inconsistently, there was no significant difference in aspect of CRP level on PICU admission between nonsurvivors and survivors in the present study. The prognostic value of CRP in pediatric patients with sepsis needs further investigation, especially in the aspect of the top level of CRP within 1 week after PICU admission or the changes of CRP during hospitalization.

In our present study, the incidence rate of sepsis-associated AKI was 4.9% in the training set or 5.3% in the validation set. In a recent retrospective study, 253 (18.4%) out of 1377 PICU patients developed AKI, and the most common etiologies of AKI were sepsis (76.9%) [21]. Until now, there is still no consistent report about the incidence rate of AKI induced by sepsis in children. We suspected that the low ratio of AKI and higher ratio of liver injury in our study might result from the inclusion and exclusion criteria focusing on patients with initial coagulation test and the high rate of patients with respiratory failure (37.6%). It is important that fibrinogen level on PICU admission is also associated with the occurrence of complication of AKI, liver injury, respiratory failure, or shock, especially of AKI. It is needed to pay more attention to the roles of fibrinogen involved in organ dysfunction besides liver injury.

There are several limitations in this study. First, this is a database study based on one-center PICU. Second, there was a lack of the records about the changes in fibrinogen during hospitalization; the value of changes in fibrinogen in predicting the outcome of sepsis should be further investigated. Third, the diagnosis of DIC was lacking in the present study. It is unknown whether fibrinogen is a more specific prognostic predictor in patients with sepsis-associated DIC. Fourth, the relationship between fibrinogen levels and sepsis-associated consciousness impairment or following intellectual influence was missing due to no related records. Fifth, patients being selected with coagulopathy testing on PICU admission might bring selective bias, which might contribute to most patients with normal level of WBC, high ratio of sepsis-associated liver injury, and low ratio of AKI. The conclusion needs to be confirmed in a prospective observational study with a larger population.

## 5. Conclusions

Fibrinogen is a valuable prognostic biomarker for pediatric sepsis. The level of fibrinogen lower than 2 g/L on PICU admission is closely related to the greater risk of hospital death in pediatric sepsis.

## Figures and Tables

**Table 1:** Baseline characteristics of patients with sepsis in the training set.

Characteristics	Total (*n* = 819)	Survivor (*n* = 720)	Nonsurvivor (*n* = 99)	*P*
Age (month)	26.2 (8.9, 64)	26.5 (9.5, 62.6)	22 (5.8, 67.6)	0.479
Gender (male, %)	505 (61.7)	454 (63.1)	51 (51.5)	0.027
PRISM III	7 (7, 10)	7 (7, 10)	15 (12, 16)	<0.001
*Complications*				
Respiratory failure, *n* (%)	308 (37.6)	248 (34.4)	60 (66.7)	<0.001
Shock, *n* (%)	138 (16.8)	104 (14.4)	34 (34.3)	<0.001
Gastrointestinal disorder, *n* (%)	36 (4.4)	27 (3.8%)	9 (9)	0.015
Liver injury, *n* (%)	167 (20.4)	139 (19.3)	28 (28.3)	0.038
Acute kidney injury, *n* (%)	40 (4.9)	31 (4.3)	9 (9.1)	0.038
Heart failure, *n* (%)	34 (4.2)	26 (3.6)	8 (8.1)	0.037
Immune disorder, *n* (%)	61 (7.4)	55 (7.6)	6 (6.1)	0.575
Brain injury, *n* (%)	43 (5.3)	32 (4.4)	11 (11.1)	0.005
*Extracorporeal life support*				
Artificial respiration, *n* (%)	306 (37.4)	233 (32.4)	73 (73.7)	<0.001
CRRT, *n* (%)	88 (10.7)	61 (8.5)	27 (27.3)	<0.001
ECMO, *n* (%)	7 (0.85)	4 (0.6)	3 (3.0)	0.012
*Length of hospital stay* (*day*)	13 (8, 21)	13 (9, 21)	8 (4, 21)	<0.001

PRISM III = Pediatric Risk of Mortality III. Results are presented as *n* (%) or median (interquartile range (IQR)).

**Table 2:** The laboratory parameters of patients with sepsis in the training set.

Parameters	Total (*n* = 819)	Survivors (*n* = 720)	Nonsurvivors (*n* = 99)	*P*
CRP (mg/L)	55 (20, 125)	55 (20, 117)	60 (19, 165)	0.265
Lac (mmol/L)	2.2 (1.4, 3.4)	2.05 (1.3, 3.1)	3.7 (2.5, 6.6)	<0.001
PT (s)	12.6 (11.7, 13.9)	12.6 (11.7, 13.7)	13.2 (12, 16.1)	0.005
INR	1.12 (1.03, 1.22)	1.12 (1.03, 1.21)	1.17 (1.03, 1.38)	0.036
APTT (s)	34.5 (29.7, 40.9)	34.3 (29.4, 40.6)	37.1 (30.8, 46.6)	0.004
Fib (g/L)	3.1 (2.14, 4.18)	3.2 (2.27, 4.25)	2.02 (1.48, 3.06)	<0.001
ALT (U/L)	22 (14, 36)	21 (14, 34)	29 (17, 64)	<0.001
TBIL (*μ*mol/L)	7.27 (4.7, 11.91)	7.34 (4.7, 11.8)	7.01 (4.75, 14.24)	0.523
DBIL (*μ*mol/L)	2.1 (1.31, 3.67)	2.1 (1.32, 3.52)	2.23 (1.2, 4.82)	0.285
LDH (U/L)	492 (335, 850)	485 (334, 831)	547 (366.5, 1057)	0.100
*γ*-GT (U/L)	16 (11, 30)	14.5 (11, 28)	24 (14, 43)	<0.001
Cr (*μ*mol/L)	26 (20, 34)	25 (20, 33)	30 (20, 44)	0.005
BUN (mmol/L)	3.4 (2.5, 4.7)	3.3 (2.4, 4.4)	4.5 (3.1, 8.5)	<0.001
CK-MB (U/L)	22 (14, 41)	21 (13, 38)	42 (19.5, 79.5)	<0.001
PLT (×10^9^/L)	219 (119, 305)	226 (132.5, 309)	113 (29, 225)	<0.001
WBC (×10^9^/L)	6.25 (4.59, 8.74)	6.25 (4.64, 8.63)	6.49 (3.94, 10.52)	0.815

CRP: C-reactive protein; Lac: lactate; PT: prothrombin time; INR: international normalized ratio; APTT: activated partial thromboplastin time; Fib: fibrinogen; ALT: alanine aminotransferase; TBIL: bilirubin; DBIL: direct bilirubin; LDH: lactate dehydrogenase; *γ*-GT: *γ*-glutamyl transpeptidase; Cr: creatinine; BUN: blood urea nitrogen; CK-MB: creatine kinase-MB; PLT: platelet; WBC: white blood cell. Results are presented as median (interquartile range (IQR)).

**Table 3:** Univariate and multivariate *logistic* regression about laboratory parameters in patients with sepsis.

Parameters	Training set (*n* = 819)					Validation set (*n* = 454)				
	Univariate *logistic* analysis		Multivariate *logistic* analysis				Univariate *logistic* analysis		Multivariate *logistic* analysis	
	OR (95% CI)	*P*	OR (95% CI)	*P*			OR (95% CI)	*P*	OR (95% CI)	*P*
*Complications*										
Respiratory failure	1.439 (1.235, 1.676)	<0.001	1.338 (1.137, 1.574)	<0.001			1.626 (1.299, 2.036)	<0.001	1.426 (1.056, 1.925)	0.020
Shock	1.471 (1.236, 1.751)	<0.001	1.292 (1.066, 1.566)	0.009			1.894 (1.523, 2.354)	<0.001		
Gastrointestinal disorder	1.396 (1.007, 1.935)	0.046					2.149 (1.638, 2.820)	<0.001	1.454 (1.014, 2.086)	0.042
Acute kidney injury	1.433 (1.054, 1.947)	0.022					2.403 (1.674, 3.448)	<0.001	1.766 (1.127, 2.767)	0.013
Heart failure	1.475 (1.071, 2.033)	0.017					2.012 (1.355, 2.986)	<0.001	1.626 (1.007, 2.624)	0.047
Disturbance of consciousness	1.627 (1.219, 2.171)	0.001	1.580 (1.165, 2.144)	0.003			3.829 (2.592, 5.657)	<0.001	3.342 (2.164, 5.161)	<0.001
*Biochemical parameters*										
Lac	1.417 (1.302, 1.542)	<0.001	1.346 (1.217, 1.489)	<0.001			1.487 (1.329, 1.664)	<0.001	1.274 (1.126, 1.442)	<0.001
PT	1.110 (1.057, 1.165)	<0.001					1.183 (1.094, 1.280)	<0.001		
APTT	1.017 (1.008, 1.025)	<0.001					1.008 (1.001, 1.016)	0.028		
Fibrinogen	0.586 (0.488, 0.703)	<0.001	0.767 (0.628, 0.937)	0.009			0.412 (0.315, 0.538)	<0.001	0.619 (0.435, 0.880)	0.008
DBIL	1.014 (1.005, 1.023)	0.002					1.026 (1.010, 1.042)	0.002		
*γ*-GT	1.003 (1.000, 1.005)	0.040					1.009 (1.004, 1.014)	<0.001		
BUN	1.059 (1.026, 1.092)	<0.001					1.203 (1.090, 1.327)	<0.001		
PLT	0.994 (0.992, 0.996)	<0.001					0.990 (0.987, 0.993)	<0.001		

Lac: lactate; PT: prothrombin time; APTT: activated partial thromboplastin time; DBIL: direct bilirubin; *γ*-GT: *γ*-glutamyl transpeptidase; BUN: blood urea nitrogen; PLT: platelet.

**Table 4:** Baseline characteristics of patients with sepsis admitted to PICU in the validation set.

Characteristics	Total (*n* = 454)	Survivor (*n* = 400)	Nonsurvivor (*n* = 54)	*P*
*Sources*				
Surgical department, *n* (%)	23 (5.1)	19 (4.8)	4 (7.4)	0.411
Emergency, *n* (%)	345 (76)	311 (77.8)	34 (63)	0.012
Others, *n* (%)	97 (21.4)	80 (20)	17 (31.5)	0.053
*Complications*				
Respiratory failure, *n* (%)	219 (48.2)	175 (43.8)	44 (81.5)	<0.001
Shock, *n* (%)	124 (27.3)	88 (22)	36 (66.7)	<0.001
Immune disorder, *n* (%)	94 (20.7)	83 (20.8)	11 (20.4)	0.948
Liver injury, *n* (%)	94 (20.7)	69 (17.3)	25 (46.3)	<0.001
Acute kidney injury, *n* (%)	24 (5.3)	12 (3.0)	12 (22.2)	<0.001
*Supplementary treatments*				
Artificial respiration, *n* (%)	206 (45.4)	159 (39.8)	47 (87)	<0.001
CRRT, *n* (%)	67 (14.8)	44 (11)	23 (42.6)	<0.001
ECMO, *n* (%)	13 (2.9)	9 (2.3)	4 (7.4)	0.033

**Table 5:** ROC analysis of fibrinogen level for hospital mortality and sepsis-associated organ dysfunction.

Parameters	Cutoff	AUC (95% CI)	Sensitivity	Specificity
Hospital mortality	2.46	0.780 (0.711-0.850)	66.67%	82.00%
Acute liver injury (ALI)	2.79	0.606 (0.539-0.674)	46.81%	73.33%
Acute kidney injury (AKI)	2.64	0.755 (0.634-0.876)	70.83%	75.35%
Respiratory failure (RF)	3.03	0.670 (0.620-0.720)	53.88%	77.45%
Shock	3.2	0.678 (0.621-0.734)	64.52%	67.27%

## Data Availability

All data generated or analyzed during this study are available from the corresponding author.

## Abbreviations

ALT: Alanine aminotransferase
BUN: Blood urea nitrogen
Cr: Creatinine
CRP: C-reactive protein
DBIL: Direct bilirubin
Fib: Fibrinogen
*γ*-GT: *γ*-Glutamyl transpeptidase
INR: International normalized ratio
Lac: Lactate
LDH: Lactate dehydrogenase
PICU: Pediatric intensive care unit
PLT: Platelet
PRISM III: Pediatric Risk of Mortality III
PT: Prothrombin time
TBIL: Bilirubin
WBC: White blood cell.

## Additional Points

*Highlights*. Plasma fibrinogen is significantly associated with lactate level and hospital mortality in children with sepsis. Plasma fibrinogen is a valuable prognostic biomarker for pediatric sepsis. The level of fibrinogen lower than 2 g/L at the time of PICU admission is closely related to the greater risk of hospital death in pediatric sepsis.
