# Corrigendum to “Fibrinogen as a Prognostic Predictor in Pediatric Patients with Sepsis: A Database Study”

**DOI:** 10.1155/2021/5290296

**Published:** 2021-02-13

**Authors:** Xiaomeng Tang, Lujing Shao, Jiaying Dou, Yiping Zhou, Min Chen, Yun Cui, Yucai Zhang, Chunxia Wang

**Affiliations:** ^1^Department of Critical Care Medicine, Shanghai Children's Hospital, Shanghai Jiao Tong University, Shanghai 200062, China; ^2^Institute of Pediatric Critical Care, Shanghai Jiao Tong University, Shanghai 200062, China; ^3^Department of Information Technology, Shanghai Children's Hospital, Shanghai Jiao Tong University, Shanghai 200062, China

In the article titled “Fibrinogen as a Prognostic Predictor in Pediatric Patients with Sepsis: A Database Study” [1], there was an error in the abstract where the following values (0.616 [95% CI: 0.457-0.829], *P* = 0.001; 1.397 [95% CI: 1.245-1.569], *P* < 0.001, respectively) should be corrected to (0.619 [95% CI: 0.435-0.880], *P* = 0.008; 1.274 [95% CI: 1.126-1.442], *P* < 0.001, respectively).

The author explained that the error was introduced during manuscript preparation and confirms that this does not affect the results and conclusions of the article.
